# Feasibility and acceptability of the menstrual cup for non-surgical management of vesicovaginal fistula among women at a health facility in Ghana

**DOI:** 10.1371/journal.pone.0207925

**Published:** 2018-11-28

**Authors:** Gabriel Y. K. Ganyaglo, Nessa Ryan, Joonhee Park, A. T. Lassey

**Affiliations:** 1 Department of Obstetrics and Gynaecology, Korle Bu Teaching Hospital, Accra, Ghana; 2 Department of Obstetrics and Gynecology, New York University Langone Medical Center, New York, New York, United States of America; 3 College of Global Public Health, New York University, New York, New York, United States of America; 4 NYU-HHC Clinical and Translational Science Institute, New York University, New York, New York, United States of America; 5 Department of Obstetrics and Gynaecology, School of Medicine and Dentistry, College of Health Sciences, University of Ghana, Legon, Ghana; Medical University of Vienna, AUSTRIA

## Abstract

**Objective:**

To assess the feasibility of the menstrual cup for short-term management of urinary leakage among women with vesicovaginal fistula (VVF)

**Methods:**

A repeated measures design compared volume of leakage with and without the cup via a 2-hr pad test among women with VVF seeking surgical repair at a health facility in Ghana. Subsequently a gynecological exam was administered to assess safety outcomes, followed by a questionnaire to assess acceptability and perceived efficacy. A paired t-test was used to analyze reduction of leakage in ml, and percent reduction was reported. Study limitations include observer bias while evaluating adverse outcomes and the possibility of social desirability bias during questionnaire administration that might overestimate the effect of the cup and women’s acceptability.

**Results:**

Of the 32 patients screened, 11 were eligible (100% consent rate). At baseline, mean (±SD) leakage in ml was 63.2 (±49.2) (95% CI: 30.2–96.3) over two hours, while the mean leakage over two hours of use of the cup was 16.8 (±16.5) (95% CI: 5.7–27.9). The mean difference of 46.4 (±52.1) ml with use of the cup (95% CI: 11.4–81.4) was statistically significant (p = 0.02). With the cup, women experienced an average 61.0% (±37.4) (95% CI: 35.9–86.2) leakage reduction, a difference 10/11 users (91.0%) perceived in reduced leakage. One participant, reporting four previous surgical attempts, experienced a 78.7% leakage reduction. Acceptability was high—women could easily insert (8/11), remove (8/11), and comfortably wear (11/11) the cup and most (10/11) would recommend it. No adverse effects attributable to the intervention were observed on exam, although some women perceived difficulties with insertion and removal. Data collection tools were appropriate with slight modification advised.

**Conclusion:**

A larger trial is warranted for a more robust evaluation of the menstrual cup for management of urinary leakage due to VVF among women who have not yet accessed surgery or for whom surgery was not successful.

## Introduction

Vesicovaginal fistula (VVF) is a debilitating maternal morbidity that largely results from complications of prolonged, obstructed labor when the trapped fetal head applies direct pressure to pelvic tissues. This can cause widespread ischemia, tissue necrosis, and formation of a hole or fistula between the bladder and the vagina (although recto-vaginal fistula, or RVF, can also form between the vagina and rectum, this more severe, less common form is not the focus of this work) [1]. Women living with VVF experience urinary incontinence which causes discomfort, malodor, and skin irritation. They also suffer social and psychological consequences that increase their vulnerability to the negative effects of stigma thus reducing their quality of life [2]. Obstetric fistula affects one to two million women globally, disproportionately impacting those in sub-Saharan Africa and S.E. Asia, and about 50,000 to 100,000 incident cases develop each year [1, 3]. The West African nation of Ghana is estimated to have over 1,300 new cases annually [4].

Traditional management requires surgical repair [5]; however, most women either do not access surgery, or access is delayed due to various individual, social, or structural barriers [6]. When surgery is accessed, repair outcomes are variable: reported rates of fistula closure vary widely (41–100%) [3], with an average of 85% [7]. In Ghana, the estimated surgical success rate is 73% [4]. Individuals with fistula, having few or no self-management options to control the constant urinary leakage, often spend significant time and resources on compulsive cleaning of self and clothing, using found or low-cost materials to fashion absorptive tools that may ultimately exacerbate suffering [8].

While surgical management is the gold standard, the menstrual cup may be a useful option for non-surgical fistula management among women who are poor surgical candidates or who cannot access surgery. This flexible reservoir cup is efficacious in preventing leakage of menstrual blood and in eliminating odor. Although more available in Europe and North America, this device is acceptable and safe for menstrual hygiene management (MHM) among women and girls in fistula-endemic nations [9]. Programmatic and clinical case reports suggest utility of the menstrual cup for collection or control of urine leakage in women with VVF [10–12]; however, no systematic evaluation of the cup within a VVF-endemic setting has been conducted.

This study aimed to assess the feasibility (primarily efficacy, safety, and user acceptability) of using the menstrual cup over a short period among women seeking care for VVF in a clinical setting and, unlike prior reports, included user perspectives and standardized measures of leakage. Additional outcomes related to feasibility of studying the intervention [13], including rates of enrollment and consent as well as appropriateness of data collection tools, were examined to inform a larger clinical trial.

## Materials and methods

This was a single arm, non-randomized, repeated measures feasibility study of the menstrual cup for short-term non-surgical management of urinary leakage associated with VVF. Recruitment occurred from 14^th^ June to 30^th^ November 2016 and data collection was conducted on 8-9^th^ August and 2-3^rd^ December 2016 at Mercy Women’s Catholic Hospital in Ghana. Approval was obtained from the Ethical and Protocol Review Committee of the School of Medicine and Dentistry, College of Health Sciences, University of Ghana, Legon (protocol number CHS-Et/M.9-P3.2/2015-2016) on 13^th^ June 2016. The trial was voluntarily registered on clinicaltrials.gov (Clinical trial registration: NCT03414060) after enrollment began. This was done as soon as the study team became aware of this option for a small, non-randomized clinical trial to test a prototype device where the primary outcome measure relates to feasibility and secondary outcomes relate to health outcomes [14]. The full trial protocol can be accessed as a supplementary document (S4 File). The authors confirm that all ongoing and related trials for this intervention are registered.

During a VVF preoperative clinic, patients were screened, consented, and enrolled by trained study personnel (S2 File). All participants who were enrolled provided informed consent via a consent form (fingerprint or signature). As the intervention could benefit women with unrepaired fistula located high in the vagina, women were examined by a study physician (fistula surgeon) who confirmed the stage of VVF (Goh type I or II) [15] and vaginal capacity to accommodate the device. The Goh classification system is based on specific measurements, position of the fistula using fixed anatomical reference points, and possible surgical and postoperative sequelae. Depending on the distance of the lower edge of the fistula from the opening of the urethra, there are four types of fistula: Goh type I and II are high up in the vagina, whereas type III and IV are in the mid and lower vagina, respectively. Participants expressed willingness to insert and remove the device, and those with RVF or severe vaginal scarring were excluded. Without evidence from any previous application of the menstrual cup for reduction of urine leakage, a sample size calculation was developed within the study protocol that assumed a 50% reduction in leakage with use. Preliminary evaluation based on initial clinical observation and expert opinion suggested that a 65% reduction was a more appropriate estimate. With 80% power and an alpha of 0.05, a total of 11 participants were required.

### Intervention

The menstrual cup (DivaCup, Diva International Inc., 222 McIntyre Drive, Kitchener, Ontario, Canada N2R 1E8) is approved by the United States Food and Drug Administration for the collection of menstrual blood. Made of 100% silicone and holding up to 30ml, it is available as model one for nulliparous women under 30 years old and model two for multiparous women and/or women age 30 or older. All study participants used model two. Participants were taught hand hygiene, vaginal placement, removal techniques, and cup washing. They had ample opportunity to practice and were encouraged to ask questions [16]. Participants were counselled to drink enough water to allow the free flow of colorless, odorless urine, and optimal hydration status was confirmed with a color chart [17]. Women were provided with equal quantities of food. Intervention use began concurrently between participants who were enrolled together. All instruction, as well as data collection, occurred in the local language of the participant’s choice.

### Data collection and analysis

Feasibility was assessed primarily by examining the limited-efficacy [18] at reducing urine leakage, safety, and user acceptability of the device after two hours of use. Volume of urine leaked was measured via a 2-hour pad test [19]. At baseline, participants wore sanitary pads within disposable underwear for two hours without physical activity restrictions. Each pad was weighed, and the dry weight subtracted from the wet weight to obtain the baseline volume (in ml) of urine leaked. Participants then inserted the menstrual cup for two hours, and the pad test was repeated. After the cup and pads were removed, a non-study clinician examined the vaginal mucosa to assess safety outcomes, including erythema, edema/induration, erosion, and bleeding. Subsequently, participants were read a structured questionnaire (S1 File) on demographics, perceived usual severity of leakage, perceived efficacy, and acceptability (comfort while wearing; ease of insertion, removal, cleaning; interference with daily activities; whether useful to women with fistula; and willingness to recommend to others). Additional feasibility factors were assessed throughout to inform the necessary steps of a future study, including rates of enrollment and consent and appropriateness of data collection.

Data were analyzed using Stata v.13 [20]. Independent and dependent variables were examined using univariate analyses to assess central tendency, normality, and distribution. The volume leaked at baseline and with the cup inserted were compared using a paired t-test, and a 95% confidence interval was generated for the mean difference. Safety outcomes (both those observed by clinician or perceived by participant) were reported as categorical events with a description of the adverse event and associated sequelae. Acceptability outcomes were reported as the proportion of participants who agreed with aspects of acceptability. Likert-type responses of agreement (1–5) were recoded to binary responses where strongly disagree, slightly disagree, and neutral were recoded to disagree, while slightly and strongly agree were recoded to agree (except the interference with daily activities question, which was coded reversely).

## Results

The mean age of participants was 43.6 (±12.3) years; notably, this study did not include young girls (Table 1). About half (6/11) completed at least primary education and were married or co-habitating with a partner (5/11). Few (2/11) were living alone and a majority (8/11) were currently working, predominantly in unskilled labor. Participants had 2.9 (±2.1) births on average. The duration of fistula ranged from 3 months to 32 years, with a median and mean of 3.3 and 9.1 years, respectively (Table 2). About half (6/11) of the participants had previously undergone surgical repair—with one participant reporting four previous attempts.

**Table 1:** Baseline demographic characteristics of participants (N = 11).

	n (%)
**Age, years**	
Mean (±SD)	43.6 (12.3)
Min	32
Max	75
**Highest Education Completed**	
None	5 (45.5)
Elementary/primary school	4 (36.4)
Junior high school	2 (18.2)
**Marital status**	** **
Single	3 (27.3)
Married	3 (27.3)
Separated but not divorced	1 (9.1)
Divorced	2 (18.2)
Co-habitating	2 (18.2)
Widowed	0 (0.0)
**People they live with**	
Alone	2 (18.2)
With partner	4 (36.4)
With partner and other family members	1 (9.1)
With non-family members	1 (9.1)
With other family members	3 (27.3)
**Type of residence**	
Rural	6 (54.5)
Urban	5 (45.5)
**Occupation**	
Unskilled	7 (55.6)
Semi-skilled	1 (9.1)
Skilled	0 (0.0)
Not currently working	3 (27.3)
**Financial support for fistula treatment**	
Personal income	2 (18.2)
Donation from family	5 (45.5)
Donation from charity or organization	2 (18.2)
Borrowed money	1 (9.1)
Unknown	1 (9.1)

**Table 2:** Baseline clinical characteristics of participants (N = 11).

Parity	
**(**Mean (±SD)	2.91 (2.1)
**Previous deliveries**	
Vaginal **(**Mean (±SD)**)**	2.09 (1.6)
C-section **(**Mean (±SD)**)**	0.73 (0.9)
**Duration of fistula**	
Mean (±SD)	9.10 (10.7) years
Median (IQR)	3.25 (2–20) years
Min	3 months
Max	32 years
**Goh stage of fistula**	
Type I	10 (90.9)
Type II	1 (9.1)
**Previous surgical attempt**	
0	6 (54.6)
1	5 (83.3)
4	1 (16.7)

### Feasibility

**Fig 1:**
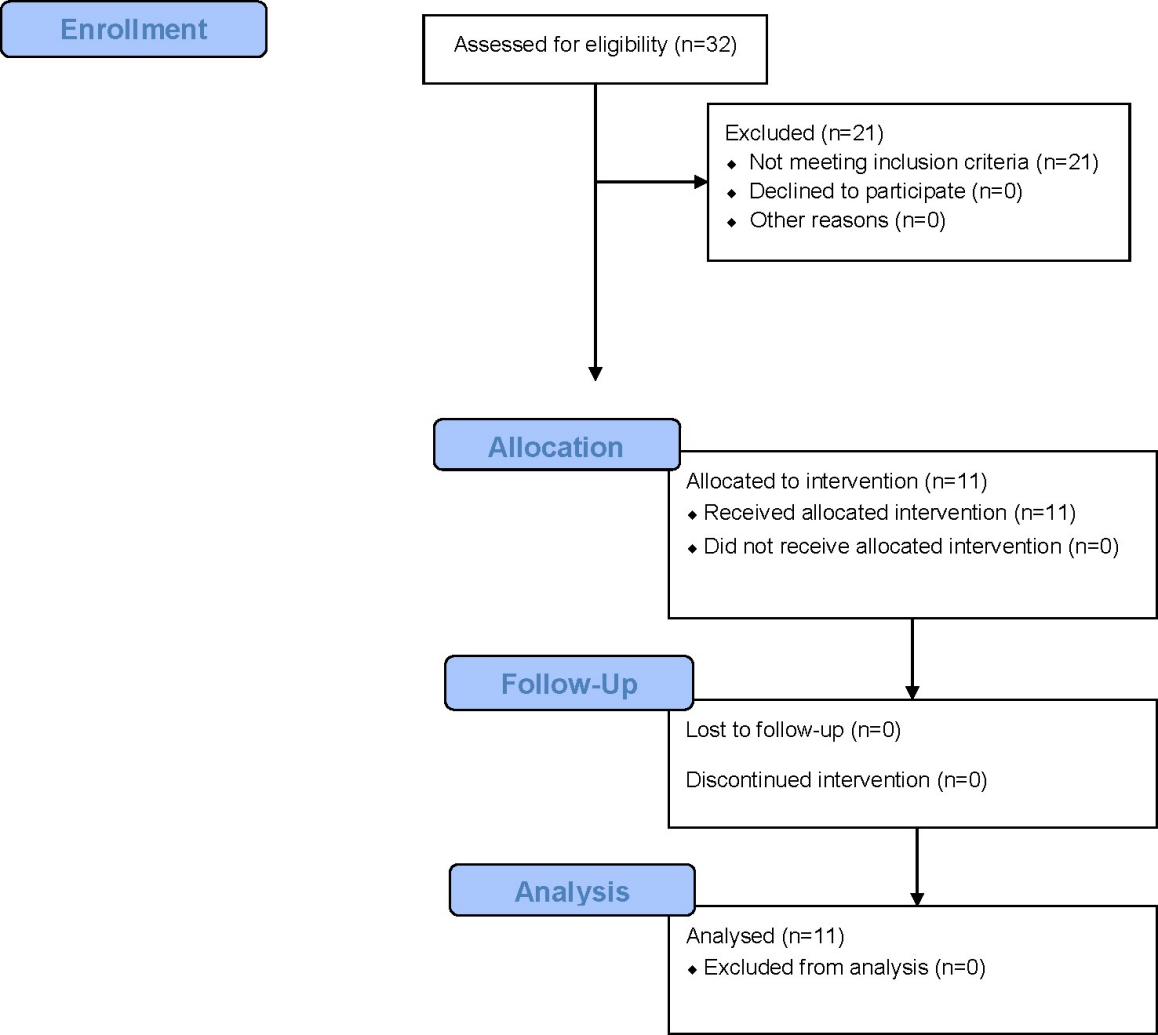
Study flow chart. The mean (±SD) volume of urine leaked over two hours at baseline was 63.2 (±49.2) ml (95% CI: 30.2–96.3). Most participants self-reported usually changing their cloth more than three times daily, with half (5/11) reporting five or more times (Table 3). The Shapiro-Wilks test suggests that the assumption of normal distribution is not rejected for observed baseline leakage (W = 0.94; critical threshold = 0.85).

Feasibility of use of the menstrual cup for short-term management of fistula was apparent, as was the feasibility of studying the intervention in this setting. Of the 32 women screened at the health facility, 11 were Goh type I or II (Fig 1). All eligible patients consented to participate. Reasons for exclusion include not having fistula (7), having Goh type 3 or 4 fistula (5), or having post-fistula repair incontinence (9). Length of time per participant to be consented, examined, and administered the questionnaire was 75–90 mins, 15 mins, and 10–20 mins, respectively, and the evaluation of the cup’s efficacy took four hours. Data collection tools, including the pad test, clinical exam, and questionnaire, were appropriately administered within this context. The trial was ended after 11 participants were enrolled and feasibility outcomes could be assessed among the sample.

**Table 3:** Observed and perceived leakage and change in leakage.

Participant ID	Number of previous attempts at surgical repair	Frequency of daily pad change^a^	Baseline leakage (ml)	Leakage with cup inserted (ml)	Reduction in leakage (ml)	Reduction in leakage (%)	Perceived reduction leakage ^b^	Indicated negative attitude or perception regarding acceptability	Perceived adverse events
1	1	—	35.6	2.7	32.9	92.4	++	Insertion; Cleaning	Heaviness in the vagina
2	0	3–5	46.6	45.4	1.2	2.6	+	Interference with daily activities	
3	0	5+	124.3	0.7	123.6	99.4	+	Removal; Not useful	
4	1	3–5	87.7	21.7	66	75.3	++		
5	0	5+	2	0.9	1.1	55.0	++		
6	0	5+	31.6	7.1	24.5	77.5	++	Removal	
7	1	5+	62	39.4	22.6	36.5	0		Couldn't void with cup inserted
8	1	3–5	29.7	33.9	-4.2	-14.1	++		
9	4	5+	98.3	20.9	77.4	78.7	++	Cleaning	
10	1	3–5	160.9	9.8	151.1	81.0	+	Insertion; Not useful	Comfortable at first, lower abdominal pain when it descended
11	0	3–5	16.6	2.2	14.4	86.7	+	Insertion; Removal	Vaginal pain; Lower abdominal pain
Mean (±SD) Median (IQR)	NA	NA	63.2 (49.2) 46.6 (29.7–98.3)	16.8 (16.5) 9.8 (2.2- 33.9)	46.4 (52.1) 24.5 (1.2- 77.4)	61.0 (37.4) 77.5 (36.5–86.7)	NA	NA	NA

^a.^ 3–5 = three to five times a day; 5+ = more than five times a day; — = don’t know

^b.^ ++ = leakage markedly improved, + = leakage slightly improved, 0 = no change in leakage, - = leakage slightly worse, — = leakage markedly worse

NA = not applicable

### Efficacy and safety

Objective and subjective efficacy of the cup is given in Table 3. A reduction in leakage was observed for 10/11 (91.0%) of participants and the same amount perceived a reduction in leakage. On average, the volume of urine leaked at baseline was reduced by 46.4 (±52.1) ml with use of the cup (95% CI: 11.4–81.4). The paired t-test suggests with 95% confidence that the mean difference in leakage at baseline and with use of the cup is statistically significantly different from zero (p = 0.015). This translates into a mean 61.0% (±37.4) reduction in leakage (95% CI: 35.8–86.2). The mean reduction for those who had previously attempted surgical repair was 58.3% (±40.3), for those who had not, 64.3% (±38.1). One participant, who reported four previous surgical attempts, experienced a 78.7% reduction in leakage. Four participants perceived adverse events attributable to use of the cup over two hours, including problems voiding while the cup was inserted, pain with insertion or removal, and ‘heaviness in the vagina.’ No adverse events attributable to the intervention were observed with post-use gynecological exam.

### Acceptability

Most could easily insert (8/11) and remove (8/11) the cup. All felt comfortable while wearing the cup (Table 4) and most (10/11) would recommend it for other fistula patients (Table 5). Some discordance occurred between reported acceptability, observed efficacy, and perceived efficacy—that is, the participant found the cup acceptable and experienced an objective reduction in leakage, but did not perceive this reduction (participant #7), or she found the cup acceptable and perceived a reduction in leakage, but did not experience an objective reduction in leakage (participant #8) (Table 3). The only participant not to recommend the cup reported ‘heaviness in the vagina’ and explained that the cup ‘gets full early and needs constant removal.’ A reduction of 32.9 ml (92.4%) was observed for this participant who voided once while using the cup and reported difficulty with insertion. The only participant to report perceived interference with social activities also perceived a slight improvement in leakage which was supported with observation of 1.2 ml (2.6%) leakage reduction. The four participants who reported not being able to usually stay long at social activities (such as at the church, mosque, funerals, or market), experienced a 99.4%, 77.5%, 36.5%, and -14.1% reduction in leakage with use of the cup, respectively.

**Table 4:** Acceptability of intervention among users (N = 11).

**Measures of acceptability n (%)**	
Comfortable to wear	11 (100.0)
Perceived useful	9 (81.8)
Easy to clean	9 (81.8)
Easy to remove	8 (72.7)
Easy to insert	8 (72.7)
Interfered with daily activities	1 (9.1)
**Would use longer n (%)**	
Yes	4 (36.4)
No	7 (63.6)

**Table 5:** Participant indicated reasons for and against recommending menstrual cup to other women (N = 11).

	Freq (%)
**Yes, would recommend**	10 (90.9)
*With [the cup] in place*, *it takes long before your underwear will be wet*.	*Participant #2*
*Only if useful or beneficial to the [woman]*.	*Participant #3*
*When I used it here it helped me with the urine leakage*.	*Participant #4*
*It was useful to me*.	*Participant #5*
*It reduced the leakage*.	*Participant #6*
*It may be helpful*.	*Participant #7*
*It's easy to insert and remove*.	*Participant #8*
*It is helpful at reducing cost of buying pad*.	*Participant #9*
*If delicate no*, *use a wrapper*.^*a*^ *If not delicate*, *[then] use*. *If they leaked a lot*, *I would recommend*.	*Participant #10*
*Easy to clean and when [inside] you feel comfortable*.	*Participant #11*
**No, would not recommend**	1 (9.1)
*It gets full early and needs constant removal*.	*Participant #1*

^a.^ wrapper is a local term for material that is wrapped around a woman’s lower body

## Discussion

The use of the menstrual cup to reduce fistula-related urinary leakage and the study of this device were feasible within this setting. This was evaluated by indicators of efficacy, safety, and acceptability, while feasibility of studying the intervention was evaluated by additional outcomes that inform the process and management of a larger clinical trial. Limited-efficacy testing of the intervention was carried out to assess change in fistula-related urinary leakage over two hours. Short-term use of the menstrual cup was associated with a mean 61.0% reduction in urinary leakage, a difference that was both observed clinically and perceived by most participants. Women found the intervention acceptable, and the likelihood of adverse events, either observed by gynaecologic exam or shared through self-report, was seemingly low. Some participants reported difficulties with insertion and removal; however, this can be expected during initial use of the device as studies of the menstrual cup for MHM have shown that ease of insertion and removal increases with use and over time for first-time users [21].

These outcomes were consistent with previous non-standardized clinical observations of the menstrual cup’s efficacy among patients with non-obstetric fistula [10, 11]. Our more rigorous study [12] suggests a positive treatment effect in a fistula endemic context and, importantly, reports women’s attitudes and perceptions related to use of this innovation. We acknowledge a wide variability of the treatment estimate, which is likely influenced by both the small sample size and the study design that captures a realistic measure of leakage as women engage in non-restrictive physical activity. Baseline leakage did seem to represent a normal distribution.

In addition to the observed effect and high acceptability, feasibility is supported by the rates of enrollment and consent and appropriateness of data collection tools. Inclusion criteria are appropriate as participants with Goh type I or II fistula seem most likely to benefit from the device. Our study population was more educated, more employed, and less parous than similarly sized studies of Ghanaian women living with fistula [8, 22] as well as portrayed in larger national reports [4]. However, previous research occurred in the North of the country where the population is more rural and impoverished. Lastly, the questionnaire administered in the local language was appropriate; however, unanticipated responses were noted to inform future questionnaire iterations and qualitative components of this work.

### Strengths and limitations

A strength of this study was the repeated measures design that estimated the effect with and without the cup, where each woman experiences both control and experimental assignment. Additional strengths included standardizing hydration status, food intake, time of use, and measures of leakage. Lastly, an effort was made to capture women’s perceptions and attitudes, not just by examining their acceptability of this innovative device to addressing chronic leaking, but also through pairing subjective assessment of leakage and efficacy with objective clinical measures.

There are some limitations, including possible bias, within this work. One potential limitation was the small sample size and short duration of observation, although both are appropriate for an early stage feasibility study. Generalizability may be limited due to the fact that young girls, who may embody different physiological factors affecting use and acceptability, did not enroll. Social desirability bias may have occurred within the study when participants were asked about their acceptability of the cup. Patients awaiting surgery may want to please their clinical providers by reporting positive experiences only; however, this was mitigated by the study staff’s effort to build rapport and encourage frank responses, evidenced by the few women who expressed their negative opinions about using the cup. In addition, the potential for observer bias was addressed by the purposeful selection of a non-study clinician to examine participants after their use of the intervention.

As experiences of fistula are not uniform, so too was experience with use of the menstrual cup for fistula management. The one very small [participant 2] and one negative [participant 8] treatment effect observed could be explained by suboptimal inclusion criteria. A large fistula may require more frequent emptying of the cup, and consequently the cup would not have been able to provide the presumed reservoir function. Alternatively, there may have been a measurement error by failing to collect all of the leaked urine in participant #8. Lastly, as intended use would require women to wear the cup for longer than the two hours observed within this trial, this study is limited in that it does not address issues of long-term use.

### Implications for research and practice

This study illustrates various implications for future studies. Research may evaluate the cup among women with other types of fistula and thus increase the generalizability of results. Changes to the design of the cup to support longer-term use could be explored, such as a modification to allow the user to externally store the leaking urine until she is able to empty, clean, and reinsert the device. The intervention could be evaluated over a longer period of use and within the community setting (i.e. at home where women would use the cup daily, rather than in the health facility). Qualitative research would provide a more robust understanding of attitudes and beliefs regarding self-management of fistula and use of this device among women, as well as of acceptability among additional intervention implementers and other fistula stakeholders. Future research should include user self-report of efficacy and could examine access to appropriate water and sanitation facilities to maintain the device.

Various implications for clinical practice also are evident. For many women who develop fistula, the variable duration between onset of symptoms and access to successful surgical repair causes many to continue to leak urine [23], thus increasing vulnerability to stigma and reducing quality of life [24]. Many women with the condition in endemic regions present late to hospital or not at all [23]. The average duration of fistula in this study was 9.1 (±10.7) years and the national estimate is 7.5 (±8.9) years [4]. Due to complex, multifactorial reasons [6], treatment, care, and support for fistula patients is further delayed even after initial contact with a health facility. This study provides evidence that an insertable cup should be further evaluated for its potential effectiveness and acceptability to manage urinary leakage for women living with fistula, including those who have not yet accessed surgical repair and those for whom surgery was not successful.

## Supporting information

S1 File Structured questionnaire. Structured questionnaire was read to participants in the local language.
(PDF)

S2 File Consent form. Consent form was read to participants in the local language.
(PDF)

S3 File Statistical analysis plan. (PDF)

S4 File Protocol. (PDF)

S5 File CONSORT 2010 checklist. (DOC)
