# Supplementary material for: Feasibility and acceptability of the menstrual cup for non-surgical management of vesicovaginal fistula among women at a health facility in Ghana
Source: PLoS One. 2018 Nov 28;13(11):e0207925. doi: 10.1371/journal.pone.0207925 (PMC6261596; doi:10.1371/journal.pone.0207925)
Supplement: S1 File — Structured questionnaire was read to participants in the local language. (PDF) [file pone.0207925.s001.pdf]

VAGINAL MENSTRUAL CUP FOR SHORT TERM NON-SURGICAL MANAGEMENT OF VVF –

STUDY SUBJECT QUESTIONNAIRE

1. Date (Day/Month/Year):__________

2. Patient's Initials (optional): __________ 3.Subject ID:__________

4. Staff initials (interviewer) ________

DEMOGRAPHICS

The following questions ask about demographic characteristics (your age, who you live with and how you receive income).

Please complete questions 5-10 with the ONE best answer.

Question 11 may have more than ONE answer.

5. In what type of residence do you currently live?
   a) Rural (village)
   b) Urban (a big town)

6. With whom do you live?
   a) I live alone
   b) I live with my partner
   c) I live with my partner and other family members
   d) I live with people not in my family

7. What is your age at the time of the study? ______(years)

8. What is the highest educational level you have completed?

a) none (no formal education)
b) elementary/ primary school
c) junior high school/ O' level
d) senior high school/ A' level
e) tertiary level (post senior high training, polytechnic, university)

9. What is your marital status at the time of study?

a) single
b) married
c) separated but not divorced
d) divorced
e) co-habiting
f) widowed

10. What is your professional occupation at the time of the study?

a) student/ apprentice /learning a trade
b) house wife
c) unskilled
d) semi-skilled (e.g. seamstress, hairdresser etc.)
e) skilled
f) not currently working

11. How were you able to support the cost of travel and surgery at the time of the study? (you may select more than one answer)

a) personal income
b) donation from family/relation
c) donation from non-family community member
d) donation from charity or organization (please list) : ....
e) borrowed money, please indicate source………………………………..
f) other (please explain): ....

SEVERITY OF URINARY LEAKAGE

Please complete questions 12-13. They ask about your experience with leakage before taking part in the study.

12. How often do you change your cloth or pad in one day (approximately)?

a) once a day
b) twice a day
c) 3-5 times a day
d) more than 5 time a day

e) don’t know

13. Regarding social activities such as church, mosque, funerals, and market, which of the following applies to you?

a) I go often and I stay
b) I go often but I do not stay long
c) I go rarely
d) I never go

For option ‘b to d’ please, explain choice if reason is other than worries about urinary leakage……………………………………………………………………………………….

SUBJECTIVE ASSESSMENT OF LEAKAGE WITH MENSTRUAL CUP

Please complete questions 14-15. They ask about your experience with leakage while taking part in the study.

14. Please compare the amount of leakage you perceived with the cup to the amount of leakage you perceived without the cup.

a) Leakage markedly improved when using the cup
b) Leakage slightly improved when using the cup
c) No change in leakage
d) Leakage slightly worse when using the cup
e) Leakage markedly worse when using the cup

15. Please compare the degree of wetness experienced with the cup to the degree of wetness experienced without the Cup. (You may select only one answer)

a) Wetness markedly improved when using the cup
b) Wetness slightly improved when using the cup
c) No change in wetness
d) Wetness slightly worse when using the cup
e) Wetness markedly worse when using the cup

SUBJECT’S ACCEPTABILITY OF MENSTRUAL CUP

Please complete questions 16-24. They ask about your experience with the cup.

Please indicate your agreement with the following statements

16. The cup was easy to insert.

Strongly disagree   Slightly disagree   Neutral   Slightly agree   Strongly agree

17. The cup was comfortable while it was inside the vagina.

Strongly disagree   Slightly disagree   Neutral   Slightly agree   Strongly agree

18. The cup interfered with my activities for the day.

Strongly disagree   Slightly disagree   Neutral   Slightly agree   Strongly agree

18a. IF Slightly/strongly agree, specify ……….……………………………

19. The cup was easy to remove.

Strongly disagree   Slightly disagree   Neutral   Slightly agree   Strongly agree

20. The cup is useful for women with urinary leakage from fistula.

Strongly disagree   Slightly disagree   Neutral   Slightly agree   Strongly agree

21. The cup is easy to clean.

Strongly disagree   Slightly disagree   Neutral   Slightly agree   Strongly agree

22. Did the cup have any effects that you DID NOT like? (Allow subject to state, do not read, circle all that apply)

a) Itch
b) Vaginal pain
c) Lower Abdominal pain
d) Vaginal bleeding
e) New odor
f) Other…………………………….

23. Would you use the cup for a longer period?

a. YES   b. NO

23a. IF YES, please indicate how long you would like to use it (if being administered by staff: listen to patients estimate first, THEN tick)

a) Several hours, but not longer
b) For an entire day, but remove at night
c) For several days, including nights
d) Other, PLEASE STATE……………………...

23b. If YES, please indicate how often you would like to use it.

a) Rarely (less than once a week)
b) Once a week or more
c) Every other day (3X a week)
d) Every day

24. Would you recommend the cup to someone in your community who is experiencing leaking urine due to fistula? Encourage patients to express their opinion freely

a. Yes, please explain…………………………..
b. No, please explain……………………………

End of questionnaire. Thank you for participating in this study.

For clinical staff to complete from patient chart or interview:

1. Date (Day/Month/Year):__________

2. Patient's Initials: __________ 3. Subject ID:__________

OBSTETRIC INFORMATION

4. № Vaginal deliveries:__ __
7. № C Sections: __ __
8. Parity:__ __
9. Goh stage of fistula: ___
10. Duration of fistula: ___(years)___(months) ___ (days)
11. № previous attempts at repair: __ __

12. Cause of Fistula: a) OBSTETRIC b) NON-OBSTETRIC

STATE OF VAGINAL MUCOSA POST CUP INSERTION

13. Erythema a) YES b) NO
14. Edema/Induration a) YES b) NO
15. Erosion a) YES b) NO
16. Bleeding a) YES b) NO
17. Other (specify) a) YES b) NO
If yes, specify:________________________________
18. Fistula size a) Increased b) Decreased c) Unchanged
