# Supplementary material for: Feasibility and acceptability of the menstrual cup for non-surgical management of vesicovaginal fistula among women at a health facility in Ghana
Source: PLoS One. 2018 Nov 28;13(11):e0207925. doi: 10.1371/journal.pone.0207925 (PMC6261596; doi:10.1371/journal.pone.0207925)
Supplement: S2 File — Consent form was read to participants in the local language. (PDF) [file pone.0207925.s002.pdf]

VAGINAL MENSTRUAL CUP (DIVACUP) FOR SHORT TERM NON-SURGICAL MANAGEMENT OF VESICOVAGINAL FISTULA—PARTICIPANT CONSENT FORM

Introduction

Fistula is a debilating condition that affects women during their reproductive ages. It primarily results from prolonged obstructed labour and lack of timely emergency obstetric care. Fistula is not caused by infidelity, witchcraft or a curse. It is a mark of failure of the health care system to provide timely care for women in labour. Once afflicted, these women are abandoned by the husbands, family and friends. They are also shunned by the community due largely to myths and misconceptions about the causes of fistula.

Some leak faeces or urine only. Others leak both urine and faeces. Once the fistula has occurred, the affected person needs surgery for cure. Unfortunately, very few surgeons have the skill to perform corrective surgery. As the numbers of patients are more than the available surgeons, the few who can perform the surgery go round the country periodically to perform the repairs. Patients continue to leak for variable periods of time before surgical correction is achieved. To help reduce the volume of leakage while the patient waits for surgery, a small plastic device called the DivaCup is being tested for use by patients who leak only urine. It may also be helpful for patients whose repair failed or were considered not suitable for surgery.

What is required from you the participant

As part of your pre-operative care, you will be examined by a gynaecologist who is also a fistula surgeon. Meals and water will be provided to you free of charge. You may leak freely but will be required to wear a sanitary pad to collect urine that you will otherwise have leaked. Sanitary pads will be provided for you free of charge.

You will be required to wear a sanitary pad for two hours. You may use more than one sanitary pad if necessary. After two hours your pad(s) will be collected and weighed.

You will be taught how to place the DivaCup in the vagina by yourself and given ample time to practice placement and removal. When you become adept at placement and removal, you will then

be asked to place the DivaCup in the vagina and wear a sanitary pad. After two hours, this pad(s) will also be collected and weighed. You will then remove the DivaCup. You will be expected to report any discomfort while wearing the DivaCup.

You will be assisted to answer a questionnaire thereafter. You have the option of filling out the questionnaire without assistance.

You are at liberty to curtail your participation at any stage during the study. Please, be assured that any information you provide would be handled confidentially and it will only be used for research purposes.

Benefit of the study to you the participant

Since your surgical repair will occur soon after your participation in this study, there is no direct benefit to you. If the DivaCup proves successful, you will obtain relief from urine leakage for the study duration. By participating in this study, you will have helped in efforts to provide short-term relief from urinary leakage for VVF patients.

Harm to you the participant

We do not anticipate any harm to you from the DivaCup. The study team has dedicated fistula surgeons who understand your plight and will do you no harm. However, in the event of pain, vaginal discharge, bleeding or any form of discomfort or distress, please, do not hesitate to notify the team immediately.

Consent

The study has been explained adequately to me and I understand that my participation is purely voluntary. I therefore give my consent and understand that I could withdraw my participation at any time without any penalty.

Name ……………………………………………………………………………………………

Right Thumb Print or Signature………………………………………

Contact Information of Investigator

If you have any further questions or for any reason you wish to withdraw your consent, you should contact me on the following address:

Dr. GABRIEL Y.K GANYAGLO

DEPT OF OBSTETRICS AND GYNAECOLOGY

KORLE BU TEACHING HOSPITAL KORLE BU, ACCRA.

EMAIL: GGANYAGLO@HOTMAIL.COM Tel: **024 4807426**
