# Supplementary material for: Feasibility and acceptability of the menstrual cup for non-surgical management of vesicovaginal fistula among women at a health facility in Ghana
Source: PLoS One. 2018 Nov 28;13(11):e0207925. doi: 10.1371/journal.pone.0207925 (PMC6261596; doi:10.1371/journal.pone.0207925)
Supplement: S3 File — (PDF) [file pone.0207925.s003.pdf]

STATISTICAL ANALYSIS PLAN

‘Feasibility and Acceptability of the Menstrual Cup for
Non-Surgical Management of Vesicovaginal Fistula among Women
at a Health Facility in Ghana’

CONTENTS

1 INTRODUCTION

Obstetric fistula, a debilitating birthing injury, leads to urinary incontinence and subsequent psychosocial and economic consequences for as many as two million women predominantly in Sub-Saharan Africa. Surgical repair is possible; however, most women either do not access surgery or successful repair is not guaranteed, leaving them few or no options to control chronic urinary leakage. The menstrual cup is a medical device inserted into the vagina to collect menstrual flow, which could also be used to control urine leakage for women with vesicovaginal fistula (VVF). While surgical management of VVF would remain the gold standard in treatment, the menstrual cup could be an alternative for women who do not have access to surgery or are poor surgical candidates. Therefore, this study will assess the feasibility of the menstrual cup for short-term management of fistula among women with VVF in a health facility in Ghana. A repeated measures design will be used to compare leakage pre and post 2-hr use of the device among 11 women.

2 OUTCOMES

Feasibility

- Efficacy at reducing urine leakage over 2-hrs measured via pad test [1]
- Safety (clinical observation of adverse events)
- User acceptability
- Enrollment rate
- Consent rate
- Appropriateness of data collection tools

Secondary

- Efficacy (subjective measure)
- Safety (subjective measure)

3 DATA SOURCE

3.1 Participants were included if they met the following criteria:

1. VVF confirmed by gynecologic examination
2. Adequate vaginal capacity to accommodate the cup
3. Fistula high in vagina (determined at gynecologic examination)
4. Willingness to insert and remove the device by one’s self

3.2 Participants were excluded if they met the following criteria:

1. Technically difficult to insert and or remove the cup e.g. severely scarred vagina

2. Unable or unwilling to learn to insert and remove cup
3. Patient who declines consent or is incapable of consent
4. Presence of rectovaginal fistula (RVF) or combined RVF and VVF
5. Fistula low in the vagina precluding collection of urine by the cup

4 ANALYSIS OBJECTIVES

This study uses a repeated measures design, whereby each woman experiences both control and experimental assignment, to assess feasibility of the menstrual cup for short-term management of fistula. Feasibility will be assessed primarily by examining the efficacy at reducing urine leakage, the cup's safety, and user acceptability of the device.

For limited-efficacy testing [2], the cup's effect will be determined by estimating the change in volume of urine leaked before and while using the cup, which will be measured via a 2-hour pad test. [1]

Post-use safety outcomes will be assessed objectively by gynecological examination (erythema, edema/induration, erosion, and bleeding) and subjectively by questionnaire responses (itch, vaginal pain, lower abdominal pain, vaginal bleeding, new odor, other).

After use, participants will be read a structured questionnaire on demographics, clinical characteristics, perceived usual severity of leakage [4], perceived efficacy, and acceptability. Using a Likert scale with values ranging from 1-5, acceptability will be reported based on ease of insertion, removal, and cleaning; comfort while wearing; useful for fistula management; interference with activities. Responses will be dichotomized where 0 will equal disagreement, while 1 will equal agreement with the respective aspect of acceptability (the exception is interference with activities which is reverse coded). A summarizing acceptability scale will not be reported.

Additional feasibility factors will be assessed throughout to inform the necessary steps of a future study, including rates of enrollment and consent and appropriateness of data collection. Appropriateness of the data collection tools will be determined by whether tools can be interpreted in local dialects and whether participants understand questions.

4 ANALYSIS SETS/ POPULATIONS/SUBGROUPS

This single-arm, unblinded clinical feasibility trial will be conducted at Mercy Women's Catholic Hospital in Mankessim in the Central Region of Ghana. Participants seeking care for obstetric fistula will be included. Both women who have not yet accessed surgery and those who have previously attempted repair will be included in the sample, and their outcomes will be compared.

Intervention

The menstrual cup (DivaCup, Diva International Inc., 222 McIntyre Drive, Kitchener, Ontario,

Canada N2R 1E8) is approved by the United States Food and Drug Administration for the collection of menstrual blood. Made of 100% silicone and holding up to 30ml, it is available as model one for nulliparous women under 30 years old and model two for multiparous women and/or women age 30 or older. All study participants will likely use model two, although this will be informed by expert clinical opinion decided upon during initial gynecological exam.

5 HANDLING OF MISSING VALUES AND OTHER DATA CONVENTIONS

Data collectors will carry out quality control efforts to ensure questionnaires are complete. If any data is missing before the end of the trial, nurses will follow up with participants. In the case that there is missing data and it is missing at random, then case-wise deletion will be used. In the case that data is not missing at random, imputation will be carried out.

6 STATISTICAL METHODOLOGY

This clinical trial will focus primarily on feasibility of the intervention by evaluating efficacy, safety, and user acceptability. Data will be analyzed using Stata v.13. Independent and dependent variables will be examined using univariate analyses to assess central tendency, normality, and distribution. The volume leaked at baseline and with the cup inserted will be compared using a paired t-test. A 95% confidence interval will be generated for the mean difference.

Sample size calculation

An a priori sample size calculation was carried out. Using Stata software and assuming a 50% reduction in leakage with use of the cup, with 90% power and an alpha of 0.05, a total of 18 subjects would be required. At the rate of about 15 fistula repairs per quarter, knowing that not all fistula patients will be eligible, the 18 subjects can be recruited in 6 months.

We originally used no estimate of variation in the original calculation that used 50% reduction for effect size and produced 18 participants needed. We later used an educated guess from a clinical expert and initial observations of effect and variance to modify the calculation using a 65% effect size and produced 11 participants needed. This calculation used alpha=0.05, power= 0.80, delta=-0.90, d0=0.00, da=-37.00, sd_d=41.00, ma1=57.00 and ma2= 20.00. As the primary objective is to investigate feasibility, not just limited-efficacy testing, the study team agreed that after 11 participants we could appropriately assess the feasibility outcomes.

Efficacy

Objective assessment

An absolute and relative reduction will be assessed.

Paired t-test

d_{mean} = Sum (leakage_baseline$_1$ - leakage_withcup$_1$) + (leakage_baseline$_1$ - leakage_withcup$_1$) / number participants

percent(diff) = (diff/ leakage_baseline) x 100

Let x = volume leaked without the cup and y = volume leaked with the cup. To test the null hypothesis that the true mean difference is zero, the procedure will be:

1. Calculate the difference ($d_i = y_i - x_i$) between the two observations on each pair.
2. Calculate the mean difference, d_{mean}.
3. Calculate the standard deviation of the differences, s_d, and use this to calculate the standard error of the mean difference, $SE(d_{mean}) = s_d \sqrt{n}$.
4. Calculate the t-statistic, which is given by $T = d_{mean}\ SE(d_{mean})$. Under the null hypothesis, this statistic follows a t-distribution with $n - 1$ degrees of freedom.
5. Use tables of the t-distribution to compare your value for T to the t_{n-1} distribution. This will provide the p-value for the paired t-test.

For this test to be valid the differences only need to be approximately normally distributed. Therefore, it would not be advisable to use a paired t-test where there were any extreme outliers. We will need to assess for outlier using the following formula [3]:

$$[Q_1 - k(Q_3 - Q_1), Q_3 + k(Q_3 - Q_1)]$$

where Q_1 is the lower interquartile range, Q_3 is the upper interquartile range, k=1.5 indicates an outlier, and k=3 indicates an extreme outlier.

To estimate a confidence interval for the true mean difference, we will use the following formula:
d_{mean} +/- (t* x $SE(d_{mean})$)

where t* is the 2.5% point of the t-distribution on n-1 degrees of freedom.

Subjective assessment
Difference in frequency of pad change while using the cup compared to baseline.

<u>Cup safety</u>

Objective assessment
Frequency of report of erythema, edema/induration, erosion, bleeding, change in fistula size after clinical examination

Subjective assessment
Frequency of self-report of abdominal pain, vaginal pain, itch, vaginal bleeding, new odor

<u>User acceptability</u>
Acceptability outcomes will be reported as the proportion of participants who agreed with aspects of acceptability. Likert-type responses of agreement (1-5) will be recoded to binary responses where strongly disagree, slightly disagree, and neutral will be recoded to disagree, while slightly and strongly agree will be recoded to agree (except the interference with daily activities question, which will be reverse coded).

<u>Demographic and clinical characteristics</u>

Demographic and clinical characteristics data will be explored using univariate analyses to describe the sample.

7 TABLES

For all tables, continuous variables will be reported with mean (SD) or with median (IQR) in the case there are outliers. Categorical variables will be reported with proportion (%) in each category.

Table 1. Demographics and clinical characteristics
Table 2. Observed and Perceived Efficacy
Table 3. Acceptability

8 QC PLANS

To ensure that the rights and well-being of the participants are protected, the study personnel will confirm that the participant has signed the consent of the subject prior to taking part in the trial and that the relevant personnel and test equipment are properly tested, including a calibrated scale. To ensure the quality of the clinical data for each participant, the data manager (NR) will check whether the questionnaire is complete or whether there is an omission.

Table 1. Study variables and descriptions

**Variable Name**	**Description for Table**
qn1	Date of enrollment
qn3	Subject ID
qn5	Residence
qn6	Who living with
qn7	Age
qn8	Education
qn9	Marital status
qn10	Occupation
qn11	Source financial support
qn11a	Source financial support (other)

qn12	Daily pad change frequency
qn13	Social interference
qn13b	Social interference explanation
qn14	Perceived effect leakage
qn15	Perceived effect wetness
qn16	Ease insert
qn17	Comfort wearing
qn18	Interference with activities
qn18b	Interference with activities_specify
qn19	Ease remove
qn20	Useful
qn21	Ease clean
qn22	Perceived adverse effects
qn22a	Perceived adverse effects (other)
qn23	Longer use
qn23a	How much longer use
qn23ai	How much longer use (other)
qn23b	Frequency of use
qn24	Recommend
qn24a	Recommend yes_explain
qn24b	Recommend no_explain

ob4	Delivery_vaginal
ob7	Delivery_csection
parity	Parity
goh	Goh stage
ob10	Duration fistula
ob11	Repair attempts
ob12	Cause of fistula
ob13	Observed adverse event_erythema
ob14	Observed adverse event_edema
ob15	Observed adverse event_erosion
ob16	Observed adverse event_bleeding
ob17	Observed adverse event_other
ob17a	Observed adverse event_other (specify)
ob18	Fistula size

10 REFERENCES

[1] Abrams P, et al., Fourth International Consultation on Incontinence Recommendations of the International Scientific Committee: Evaluation and treatment of urinary incontinence, pelvic organ prolapse, and fecal incontinence. Neurourol Urodyn, 2010. 29: p. 213–40.

[2] Bowen DJ, Kreuter M, Spring B, et al. How We Design Feasibility Studies. American journal of preventive medicine. 2009;36(5):452-457. doi:10.1016/j.amepre.2009.02.002.

[3] Tukey, John W (1977). Exploratory Data Analysis. Addison-Wesley. ISBN 0-201-07616-0. OCLC 3058187.

[4] Klovning A AK, Sandvik H, Hunskaar S. Comparison of two questionnaires for assessing the severity of urinary incontinence: The ICIQ-UI SF versus the incontinence severity index. Neurourol Urodyn 2009;28(5):411-5.
