# Supplementary material for: Feasibility and acceptability of the menstrual cup for non-surgical management of vesicovaginal fistula among women at a health facility in Ghana
Source: PLoS One. 2018 Nov 28;13(11):e0207925. doi: 10.1371/journal.pone.0207925 (PMC6261596; doi:10.1371/journal.pone.0207925)
Supplement: S4 File — (PDF) [file pone.0207925.s004.pdf]

STUDY TITLE

VAGINAL MENSTRUAL CUP FOR SHORT TERM NON-SURGICAL MANAGEMENT OF VESICOVAGINAL FISTULA (VVF)

PRINCIPAL INVESTIGATORS:

Dr. Gabriel Y.K Ganyaglo
Department of Obstetrics and Gynaecology
Korle Bu Teaching Hospital, Accra
e-mail: ggganyaglo@hotmail.com   TEL: +233 244 807 426

Veronica Ades, MD, MPH, FACOG
Department of Obstetrics and Gynecology
New York University Langone Medical Center
New York, USA
e-mail: veronica.ades@nyumc.org

OTHER INVESTIGATOR:

Joonhee Park, MD
Department of Obstetrics and Gynecology
New York University Langone Medical Center
New York, USA
e-mail: joonhee.park@nyumc.org

SUPERVISORS:

Prof. A.T Lassey

Department of Obstetrics and Gynaecology

School of Medicine and Dentistry (SMD)

College of Health Sciences

University of Ghana, Legon

Prof. S. A. Obed

Head, Department of Obstetrics and Gynaecology

SMD, College of Health Sciences

University of Ghana, Legon

CORRESPONDING INVESTIGATOR

Dr. Gabriel Y.K Ganyaglo

Department of Obstetrics and Gynaecology

Korle Bu Teaching Hospital, Accra

e-mail: ganyaglo@hotmail.com TEL: +233 244 807 426

ABSTRACT

Background

Vesicovaginal fistula is a debilitating condition resulting from prolonged obstructed labor. Globally, at least 2 million women are estimated to be living with fistula with about 50,000 to 100,000 new cases each year. More than 90% of the global burden of fistula is in sub-Saharan Africa and South East Asia. Traditional management of fistula requires surgical repair. However, many women either do not have access to surgery, or access to surgery is delayed. These categories of patients have few or no options to control the constant urinary leakage. The vaginal menstrual cup is an insertable medical device approved for use in Europe and North America to collect menstrual flow. Because it collects fluid drainage from the vagina, it could also be used to control urine leakage in women with vesicovaginal fistula. While surgical management of fistula would remain the gold standard in treatment, the menstrual cup could be an alternative for women who do not have access to surgery or are poor surgical candidates.

General Aim

To evaluate the menstrual cup (DivaCup) for short-term non-surgical management of vesicovaginal fistula.

Methodology

This study will examine the reduction in urinary leakage for women with vesicovaginal fistula who use the DivaCup over a 2 hour period. Women attending a fistula outreach will be offered enrollment prior to their surgery date. They will wear a sanitary pad for 2 hours and the pad will be weighed. The women will then receive counseling on how to insert the DivaCup in the vagina and will wear both the device and a sanitary pad for 2 hours. The second pad will also be weighed. The amount of urine leaked with and without the DivaCup will be compared using the students T-test. Additionally, the women will answer a short questionnaire on the acceptability of the DivaCup, and a physical exam will be carried out to assess safety with DivaCup use.

Expected Outcome

The knowledge gained from this study may offer alternative temporizing management options for fistulas, especially for women who fail or are awaiting surgery, women who cannot access surgery, or are poor surgical candidates.

BACKGROUND

Introduction

Vesicovaginal fistula (VVF) is an abnormal connection formed between a woman's bladder and vagina, leading to leakage of urine from the vagina (urinary incontinence).[1] Although rare, VVF in developed nations largely results from malignancy, radiation therapy or iatrogenic surgical injury.[1] However, in developing countries, VVF is a complication of prolonged, obstructed childbirth. During the prolonged labor, the trapped fetal head applies direct pressure to pelvic/vaginal tissues resulting in widespread ischemia, tissue necrosis and subsequent extensive fistula formation. [2]

Due to stigma, many women in low-resource settings with VVF do not seek treatment. As a result, the actual prevalence of VVF is unknown. Globally, it is estimated that at least 2 million women live with fistula. Each year 50,000 to 100,000 new cases are estimated to occur. This includes both vesico-vaginal and recto-vaginal fistulas (RVF).[3] A recent clinical review on obstetric fistula reported that VVF accounted for 92% to 99% of obstetric fistula cases while, RVF accounted for the remainder.[4]

A clinical review of risk factors associated with obstetric fistula reported that women in Sub-Saharan Africa and developing countries in South East Asia where access to emergency obstetric services is scarce are most commonly affected.[4] The authors additionally reported that obstetric fistula is associated with teenage status at delivery, primiparity, prolonged labor, home delivery, and short stature.[4] Wall et al however cited poverty, low social status of women, marriage at an early age, malnutrition and lack of access to emergency obstetric services as additional risk factors for VVF specifically.[1]

Women with VVF suffer from constant leakage of urine, which causes foul odor and skin infections. They also suffer divorce, separation and ostracism from their communities.[5,6] Additionally, the obstructed labor that causes the fistula often results in fetal loss in a reported 85% of cases.[6] As a result, sufferers of obstetric fistula often experience low self-esteem, feelings of rejection and depression. Loss of libido and sexual pleasure is also common among these women.[6]

Treatment for VVF depends largely on the time of presentation. In the absence of wound infection and slough formation, proponents of early repair recommend that VVF identified within 72 hours of the injury should be repaired before tissues have scarred.[2] Women with small VVF (at most 2cm in diameter) presenting within 3 months of injury can be treated with continuous bladder drainage via indwelling catheter allowing subsequent spontaneous fistula closure.[1] Unfortunately, in developing countries women often present months to years following injury, often with infections. In these cases, surgical repair is the treatment of choice but is delayed until inflammation and infection have resolved.[2]

Access to treatment requires that women have their fistula recognized by a medical professional, and be referred to a surgical center for treatment. Many women do not have access to health care, or are not able to travel far for care or afford the surgery. In developing countries, the aptitude to surgically repair VVF trails far behind the incidence. Thus, there is a large unmet need for treatment as an estimated 2 million young women live with untreated obstetric fistula in Asia and sub-Saharan Africa alone.[3] Since fistulas themselves are not usually fatal, these women are left untreated and thus live with the physical, psychosocial and economic consequences of persistent urinary incontinence.

Menstrual cup

A menstrual cup is a flexible reservoir cup that is inserted into the vagina for collection of menstrual blood. Menstrual cups have been studied since the 1950’s for their ability to improve a woman’s menstrual experience. They have been proven in several clinical trials to be sanitary, eliminate odor and prevent leakage when worn correctly. Additionally they have been shown to be comfortable, cost effective and pose no additional risk of urogenital infection.[7,8] Over the years, many manufacturers have been approved to produce these devices for the collection of blood during menstruation.

The DivaCup

The DivaCup is a brand of menstrual cup introduced to the market in 2003. It is available in two sizes. One size is intended for nulliparous women and a second, slightly larger size, is intended for multiparous women. The DivaCup is widely available and used more often in high income

countries than in middle and low income countries. It is made of 100% silicone and is flexible. The DivaCup is approved by the United States Food and Drug Administration (FDA), an agency within the United States Department of Health and Human Services whose role it is to secure the safety of medications and biologic products. This cup has been classified into Category B, Class II, which designates it as having minimal risk. The DivaCup is approved by the FDA for use for the collection of menstrual blood. This study, seeks to investigate the cup for the collection of urine in women with fistula. It will be used in women with VVF to determine its efficacy in reducing the quantity of urine leaked.

Problem statement

The UN estimates 50,000 to 100,000 cases of fistula develop globally each year, with 2 million women and girls currently living with the condition.[9] In Ghana, prevalence of obstetric fistula was estimated at 1 in 1,000 deliveries[10], with recent reports (June 2015) by the Ghana Health Service and the UNFPA suggesting between 700 to 1,300 new cases of fistula develop in Ghana each year.[11] Indeed, the global need for fistula surgery far outweighs the current capacity[12,13] and surgery remains inaccessible for 98% of the individuals currently suffering.[9] It is understood that weak healthcare infrastructure and barriers to safe delivery contributes to both the incident and prevalent burden of fistula.[1] Treatment obstacles include low number of skilled surgeons and facilities, and difficulties in recruiting rurally-isolated patients.[1]

As such, women currently suffering from fistula may benefit from a comparatively more accessible, non-surgical alternative to manage their highly stigmatizing experience of urinary leakage. The proposed device can be inserted, removed, and maintained by the fistula patient herself. This study explores the utility and acceptability of the menstrual cup as an accessible short term measure for fistula patients while they wait for surgery or potentially in the event that surgery is not successful.

Justification

In responding to a normal call to procreation, women unfortunately end with fistula and are ostracized and shunned by the community. The emotional trauma suffered by fistula patients stems from severe stigma and ostracism in their communities. The malodor from leakage of urine and/-

or faeces and the physical wetness from urine leakage serve as a barrier to intimate human contact including sexual contact that would allow women to play their expected normative function as wife, child bearer and mother. The potential for negative mental outcomes like depression and suicide is thus considerable among fistula patients.

Surgery remains the mainstay of treatment for fistula. In Ghana, fistula repairs to a large extent are performed periodically and on outreach (camp) basis due to the low number of fistula surgeons. In between camps, patients continue to leak. In the event of missing a particular camp call, or re-scheduling by the surgical team, the patient has to wait for the next camp (variable waiting time) and continues to leak. Occasionally the patient remains incontinent despite successful surgery. To alleviate the continued suffering of these patients, the DivaCup is being studied as a device for interim use while patients await definitive surgery. Without such a device, at best, patients continue to leak and wallow in misery till the next camp is due.

AIM

The overriding aim of this study is to evaluate the menstrual cup (DivaCup) for short-term non-surgical management of vesicovaginal fistula.

OBJECTIVES

Primary

To compare the volume of urine leaked with and without the DivaCup.

Secondary

1. To measure the volume of urine leaked with and without DivaCup.
2. To assess patient's comfort with use, insertion and removal of DivaCup on a likert scale.
3. To assess vaginal pain, discharge or bleeding (safety parameters) with DivaCup use

LITERATURE REVIEW

The DivaCup, created by Diva International Inc. was introduced into the market in 2003. Its founders describe the DivaCup as "a reusable, bell-shaped menstrual cup that is worn internally and sits low in the vaginal canal, collecting rather than absorbing your menstrual flow,"

reportedly allowing for 12 hours of leak-free protection.[7] [http://divacup.com/products/the-divacup/.]

The DivaCup is Canadian-designed, regulated and manufactured, but has been produced, tried and tested within International standards. Diva International Inc. is certified by the International Organization of Standardization (ISO) to manufacture the menstrual cup under medical device standards.[8] [http://divacup.com/about-us/quality-and-standards] The DivaCup was approved as an over-the-counter device by the FDA on July 16, 2002 (510k application # K021356) on the basis that it was substantially equivalent to a previously approved device – The Keeper, differing only in the material. While The Keeper was made of natural latex rubber, the DivaCup was made of liquid silicone rubber.

The product is made from medical-grade silicone, which is used in a wide variety of medical devices (eg vaginal pessaries for the management of prolapse). The molecules of liquid silicone are bound together under high heat forming a solid piece of silicone which is both flexible and durable without possibility of leaking into the body. Furthermore, the solid silicone material has been tested to ensure none of the molecules will break away from the product when boiled for cleaning. Additionally, the DivaCup does not contain latex, plastic, PVC, acrylic, acrylate, BPA, phthalate, elastomer, polyethylene, and is free of colors and dyes.[8] [http://divacup.com/about-us/quality-and-standards]

The concept of the menstrual cup was first described in the medical literature by Liswood in 1959, and was described as safe and sanitary.[14] Three years later, Karnaky et al[15] described a study of the menstrual cup in 150 women, and found that the menstrual cup was convenient and comfortable for women. They found no change in vaginal pH, no gross injury to vaginal walls, and no difference in microscopic examination of vaginal fluid.[15] Karnaky et al[15] also found no difference in vaginal bacterial flora, and a decreased incidence of *Candida albicans*.[15] Of 125 women studied, Peña et al[16] concluded that the menstrual cup was safe and effective, had no side effects of cramps, pressure, or vaginal irritation, and proved more convenient than sanitary pads.[16]

The menstrual cup has been studied for feasibility in a low-resource setting in Africa. In 2009, Averbach et al[17] introduced a product called Duet to 43 Zimbabwean women for use during menstruation. Duet is a cervical cover that can be used for contraception, STI prevention and for collection of menstrual blood. Forty-three percent (43%) of the women reported that they would use it if available, and 86% reported that it would make a difference in their lives. Concerns about the use of Duet included partner disapproval and fears that it would get stuck.[17]

A 2015 randomized clinical trial of a menstrual cup in 110 women in South Africa who were randomized to either menstrual cup or pad/tampon, found that after 3 months of use of the menstrual cup, 96% of women found it “easy to use”, 90% wanted to continue using it, and 94% would recommend it. Complaints about the menstrual cup included 10 women who reported vaginal pain with insertion. One woman in the menstrual cup group had vaginal irritation, as did one woman in the pad/tampon group. [18]

A 2011 study randomized women to DivaCup or tampons for three menstrual cycles, and measured satisfaction, comfort, infection, cost, and waste. The study found higher satisfaction among DivaCup group with 91% of women in the group stating they would continue use or recommend to a friend. Tampon use for one year was comparable in price to the cost of one DivaCup. The study was not powered to detect a difference in urovaginal infections.[19]

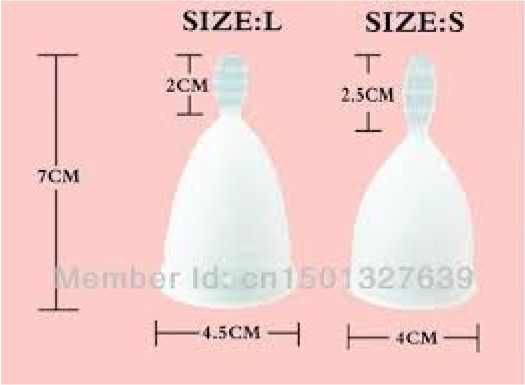

Fig. 1 left.
DivaCup with dimensions
To insert DivaCup, fold into two halves along the long axis and place fully in the vagina ensuring that no part remains too close to the introitus.

Fig 2. DivaCup without dimensions. Thumb insert is Diva 2 (for parous women) and on finger surface is Diva 1 (for nullipara)

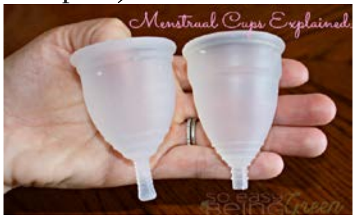

Treatment for fistula[20, 21]

Kees Waaldjik published his personal experience (1985-2008) with prolonged catheterization of fistula less than 2cm in diameter. The duration of catheterization varied between 10days to 6

weeks. He showed that up to a third can be cured on catheter alone. This however requires that the patient is seen within 75 days of the fistula occurring. In reality, a fistula can be more than 2cm in the widest diameter and most patients report later than 75 days. Surgery of varying complexity is thus the main treatment modality for fistula. In good hands, 85% of the patients can be cured on the first attempt at surgery. For a particular patient, with repeated numbers of repairs, the chances of cure dwindle.

METHODOLOGY

Study design:

This is a pre-post use cross sectional study of the DivaCup for management of urinary leakage associated with vesicovaginal fistula.

Study site

Mankessim, located in the central region of Ghana approximately 85 km west of Accra (on the Accra – Sekondi-Takoradi road) marks the beginning of the Mfantsiman district. It is located on latitude 5° 16' N and longitude 1° 01' W with a coastal savannah climate. It is bordered to the south by Saltpond, to the north by Enyan and Ajumako communities, to the east by the Ekumfi communities, and the west by Nkwanta and Krofu villages. Mankessim is about 90 minutes by road from Accra. Fishing and trading are the most popular occupations.

The Catholic Church in Ghana, with the aim of responding adequately to the needs of the disadvantaged and marginalized women, built the Mercy Women's Centre (MWC) at Mankessim in the Cape Coast Archdiocese which was commissioned in 2010. Over the 5 years of its existence, the centre has evolved into rendering general health care services for all. Currently, the centre is divided into a general care (Mercy women's general hospital) and fistula division (Mercy women's fistula centre) under one management.

The general hospital has a children's ward, a female ward, male ward, accident and emergency unit, a physiotherapy unit, records department, a theatre with a 1 bed post anaesthesia care unit, a maternity ward, labour and delivery unit, 4 consulting rooms, a pharmacy unit and a laboratory.

The fistula centre is a separate building within walking distance that has a nutrition rehabilitation unit, a mother's hostel, a laboratory, pharmacy, records unit, a spacious outpatient area, 2 consulting rooms, a spacious theatre (which accommodates 3 operating tables) with a 4 bedded post-anaesthesia care unit and a 45 bedded main ward. The fistula centre accommodates the public health unit of the hospital. The antenatal, child welfare, diabetes and hypertension clinics come under the public health unit of the hospital. The public health unit has a fistula outreach team that

goes into the communities giving health education on fistula. This team maintains a register of fistula patients and mobilizes patients for surgical repair.

With the mobilization support of the Ministry of Gender, Children and Social Protection (MOGCSP), the Mankessim centre is fast gaining stature as the national fistula centre. The MOGCSP effort brings fistula patients from all over the country to this centre. It is the most modern of the fistula units in Ghana and has the highest capacity. It has a nutrition, rehabilitation and physiotherapy units to support. Feeding and transport re-imbursement of fistula patients is routine at MWC. This is facilitated by the Cardinals Health and Education fund for the Cape Coast Archdiocese of the Catholic Church.

The Fistula Outreach team based at the KBTH makes quarterly visits to the center to repair the VVFs that have been listed for them. Other expedient visits as dictated by the arrival of a fair number of VVF patients at the center have risen in frequency with the successful mobilization efforts of the MOGCSP. Twi, Fante and English are the common languages used. However, during a MOGCSP facilitated outreach, interpreters are used for patients of northern descent.

3 resident medical officers and 60 nurses of various categories and levels form the core clinical staff. The hospital has 2 resident nurse anesthetists. During a surgical outreach, additional anesthesia support is arranged from the Korle Bu or the Cape Coast Teaching Hospitals.

Inclusion criteria

1. Patient seeking surgical repair for VVF
2. VVF confirmed by gynecologic examination.
3. Adequate vaginal capacity to accommodate DivaCup.
4. Fistula high in vagina (determined at gynecologic examination)
5. Willingness to insert and remove the device by one’s self.

Exclusion criteria

1. Technically difficult to insert and or remove the Diva cup eg severely scarred vagina
2. Unable or unwilling to learn to insert and remove DivaCup
3. Patient who declines consent or is incapable of consent

4. Presence of rectovaginal fistula (RVF) or Combined RVF and VVF.
5. Fistula low in the vagina precluding collection of urine by Divacup

Sample size determination

Using Stata software and assuming a 50% reduction in leakage using the DivaCup, with 90% power and an alpha of 0.05, a total of 18 subjects would be required. At the rate of about 15 fistula repairs per quarter, knowing that not all fistula patients will be eligible, the 18 subjects can be recruited in 6 months.

Procedures to be used

Study subjects will be identified during a VVF preoperative clinic. The subjects will be introduced to the study, and if interested in participating, the eligibility criteria will be reviewed, if eligible for enrollment, informed consent will be obtained for study participation, and the subject will be enrolled and given a subject ID.

If the number of potential patients at a single fistula camp exceeds the number required for the study, the study team will utilize randomization for enrollment. The study team member enrolling patients will ask the subjects to pick pieces of paper on which either a 1 (=invited to enroll) or 0 (=not invited to enroll) will be written. Subjects who select the pieces of paper with a 1 will be invited to enroll and given a subject ID. This will avoid selection bias in enrollment of subjects.

Once enrolled, the PI, aided by the two study team nurses will teach subjects how to place and remove the DivaCup from the vagina. A pelvic model (from the family planning unit of the hospital) will be used to demonstrate the removal and insertion techniques. They will also be taught how to wash the DivaCup using unscented soap and lukewarm water. The DivaCup can be air dried or wiped dry. They will have ample opportunity to practice removal, insertion and washing of DivaCup by themselves. The importance of hand hygiene will be emphasized. The study subjects will be encouraged to ask questions during the insertion/removal discussion.

Subjects will be given standardized sanitary pads to wear for two hours while walking around and carrying out their typical activities of daily living. It will be explained that they can use more than one sanitary pad if necessary. Each sanitary pad used in the two hours will be weighed and the dry

pad weight subtracted from the wet pad weight. Subjects will then have the DivaCup inserted. This will be followed by wearing a standardized sanitary pad for two hours. The sanitary pad will again be weighed, and the dry weight subtracted from the wet weight.

SCHEDULE OF ACTIVITIES ON DAY OF STUDY

Time	Activity			Study team
8-9am	Breakfast completed			
9-11am	Baseline leakage	Wear Pad at 9am	Remove Pad at 11am	Weigh pad
12-1pm	Lunch completed			
2-4pm	DivaCup fitted @ 2pm	Wear Pad at 2pm	-Remove pad at 4pm,<br>-followed by DivaCup<br>- Perform Post DivaCup vaginal inspection	Weigh pad
5pm	Administer questionnaire			
6pm	Serve dinner			

Quantitative assessment of volume of urine leaked

Subjects will be identified within a preoperative clinic in preparation for VVF surgery. Women with VVF will wear a standard sanitary pad for two hours. Thereafter, the pad will be weighed to measure their urinary leakage.

Women will then have the DivaCup placed, and will wear another sanitary pad for two hours. The second pad will also be weighed to estimate the volume of urine leaked. A member of the study team will weigh the sanitary pads. Quantity of urine leaked with and without the DivaCup will be compared.

Subjective assessment of volume of urine leaked

A questionnaire (appendix 1) will be administered to the women by a nurse after the DivaCup is removed. This will inquire about the severity of leakage, acceptability of the divacup and whether the patient perceived any change in the volume of urine leaked with the DivaCup.

Acceptability of DivaCup

All the women will be counselled on how to clean and place the DivaCup in the vagina by themselves. Using a subjective assessment questionnaire (see Appendix 1), they will be asked about the ease of placement and removal, comfort with use and cleaning of the DivaCup and whether they will choose to continue using it.

VVF Preoperative clinic

Prior to the date set for surgery, the patients recruited for repair are clinically reviewed. This review includes a vaginal examination to determine the type, anatomic location and size of the fistula. The nature of the vaginal tissue is assessed and the degree of scarring if any is noted. Basic laboratory investigations are also performed and the surgical approach is decided. Following this examination, patients who are candidates for DivaCup placement will be identified and informed consent obtained.

Patients are usually counselled to drink enough water to maintain colourless and odourless urine. They are encouraged to allow the free flow of urine so as to flush any disease pathogens. This is a key preoperative requirement as it minimizes the risk of infection after fistula repair. To ensure that all the eligible subjects are at their optimum state of hydration, this requirement will be emphasized.

Patient safety with DivaCup

Patients will be encouraged to report any adverse events after DivaCup placement. Notably vaginal pain, discharge or bleeding. After DivaCup removal, a vaginal inspection will be performed noting any areas of erythema, erosion, induration or mucosal injuries. Though unlikely, any changes to the fistula size will be noted. This will be documented in the patient notes. Any other unanticipated adverse events will be noted and reported.

Data handling

Data will be collected on record forms and entered into a secure REDCap (Research Electronic Data Capture) database. Data will be stripped of identifying information before entry and will be accessible only to study investigators.

Data analysis

The primary outcome will be changes in volume of urine leaked and will be determined by weighing sanitary pads worn by the subjects over two hours – without the DivaCup (as baseline), and with the DivaCup. The difference in the weights of the pads (dry weights subtracted from wet weights) will be calculated and converted to milliliters of urine leaked. The volume of urine leaked without DivaCup (before) and with the DivaCup (after) will be compared using the student's T-test. A p value < 0.05 will be set as the level of significance.

Secondary outcomes:

Subjective urinary leakage and acceptability of the DivaCup (subject approval of the method, comfort, ease of use, ease of cleaning, and whether they would chose to wear the DivaCup for a longer period of time) for management of VVF will be assessed using questionnaire in Appendix one. Questions formatted in a Likert scale will be analyzed using descriptive statistics which will be presented in tables and charts. Continuous variables will be summarized by means and standard deviations.

Any adverse safety events will be reported as categorical events, with a description of the adverse event and associated sequelae, as well as subsequent management.

DISSEMINATION OF RESULTS

The results from this study will be presented at the Obstetrics and Gynaecology departmental meetings for discussion. The results will also be presented at local and international conferences as well as published in a peer review journal.

REFERENCES

1. Wall LL. Obstetric vesicovaginal fistula as an international public-health problem. *Lancet*. 2006;368(9542):1201-1209. doi:10.1016/S0140-6736(06)69476-2.

2. Wong MJ, Wong K, Rezvan A, Tate A, Bhatia NN, Yazdany T. Urogenital fistula. *Female Pelvic Med Reconstr Surg*. 2012;18(2):71–8–quiz78. doi:10.1097/SPV.0b013e318249bd20.

3. Vangeenderhuysen C, Prual A, Ould el Joud D. Obstetric fistulae: incidence estimates for sub-Saharan Africa. *International Journal of Gynecology and Obstetrics*. 2001;73(1):65-66.

4. Tebeu PM, Fomulu JN, Khaddaj S, de Bernis L, Delvaux T, Rochat CH. Risk factors for obstetric fistula: a clinical review. *Int Urogynecol J*. 2012;23(4):387-394. doi:10.1007/s00192-011-1622-x.

5. Donnay F, Weil L. Obstetric fistula: the international response. *Lancet*. 2004;363(9402):71-72. doi:10.1016/S0140-6736(03)15177-X.

6. De Ridder D. Vesicovaginal fistula: a major healthcare problem. *Curr Opin Urol*. 2009;19(4):358-361. doi:10.1097/MOU.0b013e32832ae1b7.

7. The DivaCup | What is The DivaCup | Menstrual Cup |http://divacup.com/products/the-divacup/. Accessed September 25, 2015.

8. Quality and Standards - The DivaCup. http://divacup.com/about-us/quality-and-standards/. Accessed September 25, 2015.

9. United Nations. Report of the Secretary General, "Supporting Efforts to End Obstetric Fistula. UN doc. A/69/25 (5 Aug. 2014)

10. Danso K, Martey J, Wall L, Elkins T. The epidemiology of genitourinary fistulae in Kumasi, Ghana, 1977-1992. International Urogynecology Journal [serial online]. May 1996;7(3):117. Available from: Publisher Provided Full Text Searching File, Ipswich, MA. Accessed November 12, 2015.

11. Report on the burden of obstetric fistula in Ghana. Ghana Health Service -UNFPA report. June 2015. Page 58.

12. Polan, ML, Sleemi A, Muleta Bedane M, Lozo S, and Morgan MA. 'Obstetric Fistula' in Essential Surgery (2015) third edition of Disease Control Priorities (DCP3).

13. Browning, A., and T. L. Patel. 2004. “FIGO Initiative for the Prevention and Treatment of Vaginal Fistula.” *International Journal of Gynecology and Obstetrics* 86 (2): 317–22.14.

14. Liswood R. Internal menstrual protection; use of a safe and sanitary menstrual cup. *Obstet Gynecol*. 1959;13(5):539-543.

15. Karnaky KJ. Internal menstrual protection with the rubber menstrual cup. *Obstet Gynecol*. 1962;19:688-691.

16. Peña EF. Menstrual protection. Advantages of the menstrual cup. *Obstet Gynecol*. 1962;19:684-687.

17. Averbach S, Sahin-Hodoglugil N, Musara P, Chipato T, van der Straten A. Duet for menstrual protection: a feasibility study in Zimbabwe. *Contraception*. 2009;79(6):463-468. doi:10.1016/j.contraception.2008.12.002.

18. Beksinska ME, Smit J, Greener R, et al. Acceptability and performance of the menstrual cup in South Africa: a randomized crossover trial comparing the menstrual cup to tampons or sanitary pads. *J Womens Health (Larchmt)*. 2015;24(2):151-158. doi:10.1089/jwh.2014.5021.

19. Howard C, Rose CL, Trouton K, et al. FLOW (finding lasting options for women): multicentre randomized controlled trial comparing tampons with menstrual cups. *Can Fam Physician*. 2011;57(6):e208-e215.

20. Waaldjik K. the immediate surgical management of fresh obstetric fistulas with catheter and/or early closure. Int J Gynaecol Obstet. 1994 Apr: 45(1):11-6

21. Lassey AT. Simple fistulas: diagnosis and management in low-resource settings-a descriptive report. Int J G ynaecol Obstet. 2007 Nov; 99 Suppl 1:S47-50.

ETHICAL ISSUES

APPROVAL

This proposal will be submitted to the Ethical and Protocol Review Committee of the School of Medicine and Dentistry for approval. Permission will also be sought from the management of Mercy Women's Centre.

VOLUNTARY INFORMED CONSENT

Voluntary written informed consent will be sought from the women before inclusion in the study.

We recognize it is critical that volunteer studies be carried out in an environment where no coercion is applied and where volunteers can be adequately informed of the nature of procedures. Fistula patients are a sensitive group for human research. To ensure the informed nature of the consent, it will be administered by nurses on the study team and in the patient's language of choice. These are public health nurses with considerable experience in giving health talks to patients. The nurses are also part of the fistula case finding and recruitment team of MWC. Over the last 5 years, these nurses have worked actively with fistula patients and readily establish good rapport with each cohort of fistula patients.

The study will be introduced to all the fistula patients who responded to the camp call and in the local languages. This will enable all the women present to be informed. More detailed information will be given to those who volunteer to participate and in the particular language of their choice.

Women enrolled in this study are already seeking surgical repair for fistula, and enrolment in this study will not affect their ability to receive that treatment. The procedures employed ie drinking of water and vaginal examinations form part of the preoperative care all fistula patients receive. Additional efforts will be made to ensure the vaginal examination after study participation occurs in theatre just before the start of planned surgery. Use of the DivaCup is not expected to change the vaginal architecture or the microfloral environment as its use is for a short duration of time (2hours).

Theoretically, the DivaCup may cause vaginal erosion or pain. It is most unlikely to affect the fistula. Adverse events will be monitored, and any patient who reports an adverse event will remove the DivaCup and her participation will cease. Any adverse events will be treated appropriately. As the DivaCup insertion period is very short (2 hours), we do not anticipate any adverse events.

A breach of privacy with study data could result in the loss of privacy for subjects. We will minimize this risk by using only the subject's study ID number in all study documents. Only the consent form and the study log will contain the patient's name. These documents will be stored in locked, secured cabinets and will be accessed only by study personnel.

Because the subjects are expected to have fistula surgery soon after participation, we do not anticipate any specific direct benefit from study participation. If successful, they will have relief of their incontinence symptoms for the duration of the study period.

In addition, the subjects' participation will help the entire community of fistula sufferers by offering alternative management options for women who cannot access surgery or are poor surgical candidates.

The freedom to decline to participate or to discontinue participation at any time without penalty or alteration in the quality of due care will be made known to them. Only after this will informed consent be obtained and documented. Information for consent will be based on the attached consent form to be approved by the Ethical and Protocol Review Committee of the School of Medicine and Dentistry.

CONFIDENTIALITY

All data will be handled confidentially. Survey questionnaire and data sheets will be completed and signed by the research team only. Data will be doubly entered and checked for errors. During data entry and validation, database files will be accessible by the study investigators only, and password protected. It shall be explained to the patient that it is optional to put their name on the

questionnaire. Communication of the findings of this study would be carried out in line with the normal procedures of the School of Medicine and Dentistry.

FUNDING

The funding for this study came from a private foundation which made a research grant to the New York University (NYU) Department of Obstetrics and Gynecology. The funding and the foundation are not tied in any way to the company that makes the DivaCup. The members of the research team have no ties with Diva International Inc.

VAGINAL MENSTRUAL CUP (DIVACUP) FOR SHORT TERM NON-SURGICAL MANAGEMENT OF VESICOVAGINAL FISTULA—PARTICIPANT CONSENT FORM

Introduction

Fistula is a debilating condition that affects women during their reproductive ages. It primarily results from prolonged obstructed labour and lack of timely emergency obstetric care. Fistula is not caused by infidelity, witchcraft or a curse. It is a mark of failure of the health care system to provide timely care for women in labour. Once afflicted, these women are abandoned by the husbands, family and friends. They are also shunned by the community due largely to myths and misconceptions about the causes of fistula.

Some leak faeces or urine only. Others leak both urine and faeces. Once the fistula has occurred, the affected person needs surgery for cure. Unfortunately, very few surgeons have the skill to perform corrective surgery. As the numbers of patients are more than the available surgeons, the few who can perform the surgery go round the country periodically to perform the repairs. Patients continue to leak for variable periods of time before surgical correction is achieved. To help reduce the volume of leakage while the patient waits for surgery, a small plastic device called the DivaCup is being tested for use by patients who leak only urine. It may also be helpful for patients whose repair failed or were considered not suitable for surgery.

What is required from you the participant

As part of your pre-operative care, you will be examined by a gynaecologist who is also a fistula surgeon. Meals and water will be provided to you free of charge. You may leak freely but will be required to wear a sanitary pad to collect urine that you will otherwise have leaked. Sanitary pads will be provided for you free of charge.

You will be required to wear a sanitary pad for two hours. You may use more than one sanitary pad if necessary. After two hours your pad(s) will be collected and weighed.

You will be taught how to place the DivaCup in the vagina by yourself and given ample time to practice placement and removal. When you become adept at placement and removal, you will then be asked to place the DivaCup in the vagina and wear a sanitary pad. After two hours, this pad(s)

will also be collected and weighed. You will then remove the DivaCup. You will be expected to report any discomfort while wearing the DivaCup.

You will be assisted to answer a questionnaire thereafter. You have the option of filling out the questionnaire without assistance. You are at liberty to curtail your participation at any stage during the study. Please, be assured that any information you provide would be handled confidentially and it will only be used for research purposes.

Benefit of the study to you the participant

Since your surgical repair will occur soon after your participation in this study, there is no direct benefit to you. If the DivaCup proves successful, you will obtain relief from urine leakage for the study duration. By participating in this study, you will have helped in efforts to provide short-term relief from urinary leakage for VVF patients.

Harm to you the participant

We do not anticipate any harm to you from the DivaCup. The study team has dedicated fistula surgeons who understand your plight and will do you no harm. However, in the event of pain, vaginal discharge, bleeding or any form of discomfort or distress, please, do not hesitate to notify the team immediately.

Consent

The study has been explained adequately to me and I understand that my participation is purely voluntary. I therefore give my consent and understand that I could withdraw my participation at any time without any penalty.

Name ………………………………………………………………………………………

Right Thumb Print or Signature………………………………………

Contact Information of Investigator

If you have any further questions or for any reason you wish to withdraw your consent, you should contact me on the following address:

Dr. GABRIEL Y.K GANYAGLO

DEPT OF OBSTETRICS AND GYNAECOLOGY

KORLE BU TEACHING HOSPITAL KORLE BU, ACCRA.

EMAIL: GGANYAGLO@HOTMAIL.COM Tel: **024 4807426**

TIME LINES OF WORK

ACTIVITY	ESTIMATED TIME FRAME
ETHICAL REVIEW PROCESS	MAY TO JUNE 2016
ACTUAL STUDY (DATA COLLECTION)	JULY TO DECEMBER 2016
MANUSCRIPT	JANUARY 2017

PERSONNEL OF THE STUDY TEAM AND ROLES

NAME	BRIEF BIOSKETCH	ROLE
Dr. Gabriel Y.K Ganyaglo	7 years dedicated service as a fistula surgeon. Member of the Obstetric Fistula Care Team (OFCT) Korle Bu Teaching Hospital (KBTH). Senior Obstetrician Gynaecologist, KBTH	Special focus on protecting Patient rights, safety and autonomy. Study design, methodology, proposal and manuscript writing, data collection and data security
Dr. Veronica Ades	Assistant Professor of Obstetrics and Gynecology and Director of Global Women's Health at the New York University School of Medicine (NYUMC). Completed a fellowship in Reproductive Infectious Disease, specifically conducting NIH-funded trials of malaria prevention in Uganda. She has collaborated with Professor Samuel Obed to form an educational and research partnership between the Departments of Obstetrics and Gynecology at KBTH and NYUMC. Dr. Ades is a board-certified obstetrician-gynecologist.	Proposal and manuscript writing. Study design, methodology, data storage and analysis, data quality and security, safety.
Dr. Joonhee Park	Clinical Assistant Professor of Obstetrics and Gynecology at NYU Tisch Langone Medical Center and is a board-certified obstetrician-gynecologist. Has interest in Global Health and has participated in several volunteer gynecologic surgical mission trips to Liberia.	Funding and study supplies Proposal review.
Prof. A.T. Lassey	35 years dedicated service as a fistula surgeon. Leader of the OFCT KBTH. Associate professor of obstetrics and gynaecology, School of Medicine and Dentistry, College of Health Sciences	Advice on technical aspects of the study. Proposal and manuscript review.

Prof. S. A Obed	Head of Department of Obstetrics and Gynaecology School of Medicince and Dentistry	Administrative oversight and technical advice on study methods.
Dr. Ali Samba	15 years dedicated service as a fistula surgeon. Member of the OFCT KBTH. Obstetrician Gynaecologist, Senior lecturer Department of Obstetrics and Gynaecology, School of Medicine and Dentistry	Post DivaCup vaginal exam, post study fistula repair and oversight on adverse events
Dr. Mumuni Kareem	10 years dedicated service as a fistula surgeon. Member of the OFCT KBTH. Obstetrician Gynaecologist, Lecturer Department of Obstetrics and Gynaecology, School of Medicine and Dentistry	Post DivaCup vaginal exam, post study surgical repair and oversight on adverse events
Nessa Ryan	Nessa Ryan, MPH, PhD Candidate, New York University College of Global Public Health New York, USA	Data collection and storage. NYU IRB Liaison
Ms Rose Mantey	SRN Midwife, Public Health Nurse, with 5 years working as head of fistula recruitment and case finding team and post-operative care nurse at Mercy Women’s Centre	Patient welfare, counselling pre- and post-operative care. Demonstration of DivaCup placement and removal, questionnaire administration
Ms Dora Dankwa	Community health Nurse. 3 years working on the fistula recruitment and case finding	Patient counselling, consent form administration, demonstration of DivaCup

	team at Mercy Women's centre	placement and removal, questionnaire administration

BUDGET AND LOGISTICS

Item	Unit cost ($)	Quantity	Total Cost ($)
DivaCups	30	40	1, 200
Weighing scale	100	1	100
Sanitary Pads	4 for a pack of 24	8 packs	32
Drinking water	2	18	36
Photocopies of protocol	5	20	100
Food and Travel Reimbursement for patients	$50 per patient	40	2,000
Ethical Review	100	1	100
TOTAL			3, 568
